# Potential application of nanotechnology in the treatment, diagnosis, and prevention of schistosomiasis

**DOI:** 10.3389/fbioe.2022.1013354

**Published:** 2022-12-09

**Authors:** Abdul Qadeer, Hanif Ullah, Muhammad Sohail, Sher Zaman Safi, Abdur Rahim, Tawfik A Saleh, Safia Arbab, Petr Slama, Pavel Horky

**Affiliations:** ^1^ Key Laboratory of Animal Parasitology of Ministry of Agriculture and Rural Affairs, Shanghai Veterinary Research Institute, Chinese Academy of Agricultural Sciences, Shanghai, China; ^2^ Department of Veterinary Medicine, University of Veterinary and Animal Sciences, Lahore, Pakistan; ^3^ West China School of Nursing/West China Hospital, Sichuan University, Chengdu, China; ^4^ Key Laboratory of Molecular Pharmacology and Drug Evaluation, School of Pharmacy, Collaborative Innovation Center of Advanced Drug Delivery System and Biotech Drugs in Universities of Shandong, Yantai University, Yantai, China; ^5^ Interdisciplinary Research Center in Biomedical Materials (IRCBM), COMSATS University Islamabad, Lahore, Pakistan; ^6^ Faculty of Medicine, Bioscience and Nursing MAHSA University, Selangor, Malaysia; ^7^ Department of Chemistry, COMSATS University Islamabad, Islamabad, Pakistan; ^8^ Department of Chemistry, King Fahd University of Petroleum & Minerals, Dhahran, Saudi Arabia; ^9^ Lanzhou Institute of Husbandry and Pharmaceutical Sciences, Chinese Academy of Agricultural Sciences, Lanzhou, China; ^10^ Laboratory of Animal Immunology and Biotechnology, Department of Animal Morphology, Physiology and Genetics, Faculty of AgriSciences, Mendel University in Brno, Brno, Czechia; ^11^ Department of Animal Nutrition and Forage Production, Faculty of AgriSciences, Mendel University in Brno, Brno, Czechia

**Keywords:** schistosomiasis, praziquantel, nanotechnology, drug delivery system, liposome, nanoparticles, diagnosis

## Abstract

Schistosomiasis is one of the neglected tropical diseases that affect millions of people worldwide. Globally, it affects economically poor countries, typically due to a lack of proper sanitation systems, and poor hygiene conditions. Currently, no vaccine is available against schistosomiasis, and the preferred treatment is chemotherapy with the use of praziquantel. It is a common anti-schistosomal drug used against all known species of *Schistosoma*. To date, current treatment primarily the drug praziquantel has not been effective in treating *Schistosoma* species in their early stages. The drug of choice offers low bioavailability, water solubility, and fast metabolism. Globally drug resistance has been documented due to overuse of praziquantel, Parasite mutations, poor treatment compliance, co-infection with other strains of parasites, and overall parasitic load. The existing diagnostic methods have very little acceptability and are not readily applied for quick diagnosis. This review aims to summarize the use of nanotechnology in the treatment, diagnosis, and prevention. It also explored safe and effective substitute approaches against parasitosis. At this stage, various nanomaterials are being used in drug delivery systems, diagnostic kits, and vaccine production. Nanotechnology is one of the modern and innovative methods to treat and diagnose several human diseases, particularly those caused by parasite infections. Herein we highlight the current advancement and application of nanotechnological approaches regarding the treatment, diagnosis, and prevention of schistosomiasis.

## Introduction

Schistosomiasis is a serious tropical disease caused by parasitic flatworms of the genus *Schistosoma*, often recognized as blood flukes (Ullah et al., 2020a). Schistosomiasis is the second most prevalent parasitic disease after malaria, owing to its catastrophic impact on public health, which in turn impacts the global economy. Infection with different *Schistosoma* species may cause more intricate situations such as urinary bladder, colorectal, and liver malignancies (Li et al., 2019). *S. haematobium* infection may develop infertility in males. Testicular infarction can develop from ova occluding the spermatic venous plexus and subsequent granuloma formation (Adisa et al., 2012). As per the world health organization (WHO) data, 236.6 million individuals (WHO, 2022) in 78 countries (Ullah et al., 2022a; Ullah et al., 2022b), required schistosomiasis preventive therapy (Qadeer et al., 2021), including forty million females in the procreative phase (Cioli et al., 2014). The yearly death toll from schistosomiasis in developing nations is estimated to be over 0.3 million (Colley et al., 2014).

*S. mansoni*, *S. haematobium*, *S. Japonicum*, *S. intercalatum*, and *S. mekongi* are the five clinically important schistosomal species (Siqueira et al., 2017). The intermediate host freshwater snail infected with miracidia is needed for infection with these worms. The miracidia develop into cercaria and the cercaria penetrates the host skin after water exposure (Adekiya et al., 2020). The intermediate form produces in the aquatic gastropod snail. Generally, the intermediate host for *S. japonicum* is *Oncomelania*, *Biomphalaria* for *S. mansoni,* and *Bulinus* for *S. haematobium*. The life cycle of schistosomiasis is initiated by the exit of cercariae from the snail into the water and the penetration of the skin of humans and animals when they swim or interact with contaminated water (Gryseels et al., 2006). The adult parasites inhabit many sites of the mesenteric veins of the vertebrate host, and it is seen to be definite for every species. They are found in the venous plexus of the bladder and, on rare occasions, in the rectal venules in *S. haematobium* infection. While, adults of *S. japonicum* and *S. mansoni* are found in the superior mesenteric vein draining to the small intestine and superior mesenteric vein draining to the large intestine, respectively (Tomiotto-Pellissier et al., 2017).

Disease spread is facilitated by poor sanitary conditions as well as low economic development (Rollinson et al., 2013). The World Health Assembly passed a resolution in 2012, in Geneva, Switzerland, urging increased investment in schistosomiasis control and supporting the launch of elimination programs in endemic countries (Americas, Asia, and Africa). Schistosomiasis control and treatment are restricted to less accessible chemotherapeutic agents that show effectiveness, care, and thrmissibility (Tomiotto-Pellissier et al., 2017). Schistosomiasis is currently controlled through snail host elimination, health education, hygiene preference, clean water availability, and improved sanitation (Ferrari et al., 2003). Precise diagnosis and extensive drug administration are critical for disease management (Ullah et al., 2020b).

Praziquantel (PZQ) is the preferred treatment for schistosomiasis; it was developed in the late 1970s and is known to be an active anti-schistosomal medicine. Though, PZQ cannot treat immature worms nor avert reinfection (Dkhil et al., 2015a). Drug resistance has been observed across the world as a result of parasite mutation, inadequate tretreatment ofseases, overuse of PZQ, and mixed infection (Adekiya et al., 2020). PZQ usage is generally restricted due to its poor bioavailability and water solubility (Lindenberg et al., 2004). The metabolism leads to the formation of a less effective molecule, which must thus be given at larger doses (Mourao et al., 2005). Such factors motivate scientists, and thysician to look for technological alternatives for a safe and efficient oral absorption of PZQ, which may be utilized in disease prevention and treatment (Yang M. et al., 2009b; Ribeiro de Souza et al., 2012).

In the twenty-first century, nanotechnology emerged as one of the progressive and developing technologies. Norio Taniguchi coined the term “nanotechnology” in 1974. Nanotechnology (from the Latin nanus, which means the “dwarf”). is defined as the design, manufacture, and application of materials and technologies with he least functional content on a nanoscale (1–100 nm), or one billionth of a meter (10^−9^) (Moniruzzaman and Min, 2020). The progress of a broad variety of nanoscale technologies is the start of changing the basis of diagnosis, healthcare, and disease prevention. These technological advances are known as nanomedicines (Moghimi et al., 2005).

Nanomedicine is the application of nanotechnology to treat, monitor, and prevent diseases. For the effective use of nanomedicine, identification of the exact targets (cell and/or receptors) and precised delivery system are necessary (Abaza, 2016). Nanomedicines can play a significant role in drug delivery and disease by ensuring that a sufficient amount of the drug enters the body, that the drug does enter the body should stay for an appropriate amount of time, and that it is target-specific to the areas that require treatment (Agrawal, 2016). Nanotechnology has been used worldwide in various fields, and certain metals or metal oxides nanoparticles (NPs) are now broadly applied as drugs to cure several diseases (Elechiguerra et al., 2005; Jebali and Kazemi, 2013). Nanotechnology has the ability to restore the use of hazardous medications by the use of multifaceted structures that permit drugs to be carried to the pathogen, protecting host cells and using its impact with less toxicity (Forrest and Kwon, 2008). Previously, nanotechnology was employed as a drug delivery system (DDS) in several parasitic diseases such as malaria, toxoplasmosis, trypanosomiasis, and leishmaniasis (Date et al., 2007).

Nanotechnology and the use of nano-enabled drug delivery systems are believed to be effective in schistosomiasis treatment if combined with PZQ (Veerasamy et al., 2011). By offering more intensive drug delivery, nano-enabled drug delivery systems can improve the bioavailability, therapeutic effectiveness, and adverse effect profile of PZQ (or other medicines), (Adekiya et al., 2020). The objective of this appraisal is to emphasize and address the present state of research employing various nanoparticles as drug delivery systems that permit for drug vectorization, treatment, diagnosis, and control of schistosomiasis.

### Overview of praziquantel use as anti-schistosomal drug

In 1984, chemotherapy was suggested as the best way of treating and removing schistosomiasis by the WHO experts committee. Schistosomiasis control and treatment depend upon the single-choice treatment using PZQ. PZQ is widely used among other antischistosomal products. It is effective against all *Schistosoma* species responsible for schistosomiasis. It reduces parasitic load and can lower the intensity of symptom. Because of its easy administration, efficiency, and affordability, it is also the most preferred medication. The mechanism of action for the treatment of schistosomiasis with PZQ is not well understood, however, variations in the musculature of the worm is a suggested mechanism (Adekiya et al., 2020). Younger forms are exposed to lower levels of unchanged PZQ in the bloodstream than older forms found in the liver. PZQ is broadly changed into an inactive or significantly less effective compound after oral administration (Xiao et al., 1985; Mourao et al., 2005). In the absence of alternative schistosomicides, it is critical to implant strategies to prevent, or at least delay, the evolution of drug resistance, as well as to seek remidies to overcome some of PZQ shortcomings, such as its lack of activity on immature worms (Doenhoff et al., 2008). Due to these circumstances, scientist and clinicians were compelled to seek a safer and more effective method of treating oral absorption (Yang et al., 2009a; de Almeida et al., 2012).

Due to the shortcomings of the drugs scientists have developed drug delivery technologies (nanotechnology) to offer further focused therapies to all stages of the schistosome parasite, and the medicines may be more effective by targeting the juvenile parasites. This novel approach can also decrease drug resistance by clearing the host’s schistosome and preventing reinfection.

## Molecular targets in Schistosome tegument

The adult schistosome tegument is a type of outer surface structure composed of a cytoplasmic syncytium linked to the basal cell bodies by a thin cytoplasmic linkage. Their bodies house the nuclei, ribosomes, endoplasmic reticulum, mitochondria, and Golgi apparatus, and their vesicular products, known as discoid bodies and multilaminate vesicles, are carried to the tegument syncytium *via* the cytoplasmic connections (Pérez-Sánchez et al., 2008). The apical surface of the tegument, like the glycocalyx of eukaryotic cells, is made of a normal plasma membrane underlain by a membrane-like secretion, which has been dubbed membranocalyx (Skelly and Alan Wilson, 2006; Pérez-Sánchez et al., 2008).

This tegument, which encompasses the entire exterior of the worm, acts as an important connection between both the parasite and its host and has been vitally elucidated in the multifaceted host - pathogen interactions, which encompasses nutrient supply, excretion, osmoregulation, sensory reception, signal transduction, and interaction with the host’s immature and hemostatic systems (Van Hellemond et al., 2006; Ramajo-Hernández et al., 2007). Proteins expressed at the surface membranes of newly formed schistosomula are thus believed to be excellent candidates for the growth of novel schistosomiasis vaccines and medicines (Sotillo et al., 2015). As a result, identifying and characterizing schistosome tegumental compounds is critical for comprehending the host-parasite interaction and creating novel immunologic, therapeutic, and diagnostic methods (Skelly and Alan Wilson, 2006).

On the surface of the tegument, several targets have been identified. These are required to target schistosome glucose transporter 1 (SGTP1), schistosome glucose transporter 4 (SGTP4), acetylcholinesterase (AChE), and a nicotinic type of acetylcholine receptor (nAChR) for designed drug-loaded nanoparticles, which are mostly found on the surface of male schistosomes. Dynein, aquaporins, and tetraspanins are some of the other surface proteins present on the targeted tegument. Such compounds discovered on the surface of the tegument can be used to generate novel medicinal drugs and *Schistosoma* parasite vaccines (Adekiya et al., 2020).

### An overview of nano delivery system

Several studies have found that nano-delivery systems can improve the therapeutic effects of various substances in disease treatment (Zadeh Mehrizi et al., 2018). A nano-enabled drug delivery system can improve or modify the pharmacokinetics of active pharmaceutical ingredients (API), as well as protect API from chemical, physical, and biological degradation. However, the nano sizes of such systems improve the efficiency of biological barrier bridging, tissue tolerance, and cellular absorption and transportation, enabling for adequate delivery of therapeutic drugs to target regions such as the liver, brain, and solid tumors (Date et al., 2007; Adekiya et al., 2020). The use of solid lipid nanoparticles in the treatment of schistosomiasis would also be valuable in terms of cost-effectiveness, as they are cost-effective, comparatively less toxic, stable, and easy to decompose (Cheng et al., 2017). Lipid-based preparations can increase drug availability by changing the solubility and pace at which drugs can be freed to promote and enhance drug absorption across biological barriers. This method will be useful and successful for treating all forms of schistosomes (Adekiya et al., 2020). It has been shown that NPs can activate higher cell defense, which can prevent disease recurrence and reinfection, by specifically targeting overexpressed schistosome antigens in the human host (Tousif et al., 2017).

In *S. mansoni* infected mice, SLN-PZQ at a dose rate of 500 mg/kg showed better bioavailability, absorption rate, and antischistosomal action in all treatment groups, with a higher degree of worm reduction in all tested doses. The effective dose 95 (ED95) of solid lipid nanoparticles-PZQ was 5.29 times lower than that of market praziquantel (M-PZQ), with considerably larger intestinal tissue egg burdens and virtually total elimination of immature deposited eggs in all studied groups (Radwan et al., 2019). Another research found the greatest substantial reductions in total worm count, egg/Gram liver tissue, and intestine were seen using liposome-encapsulated PZQ at a dose rate of 500 mg/kg (97.2%, 99.3%, and 99.5% respectively). Liposomal-PZQ (Lip.-PZQ) showed greater efficacy than that of free PZQ in every aspect, particularly when administered at 45 days Post infection (Labib El Gendy et al., 2019). Table 1 lists the various nano-delivery systems utilized to improve PZQ therapeutic effectiveness in the treatment of schistosomal infections**.**

**TABLE 1:** List of different Nano-delivery systems used to improve the treatment efficacy of drug against schistosomal infections.

S. No	Nanoparticles used and dose rate	Schistosomes species studied	Model animal used in study	Efficacy	References
1	Praziquantal-solid lipid nanoparticles (PZQ-SLN) 25 and 50 µg ml^−1^	*S. mansoni*	Mice	The SLN provides a controlled release of PZQ as a higher schistosomicidal activity against *S. mansoni* culture than PZQ suspension. PZQ-SLN was found to be more successful than PZQ alone in control group, resulting in parasite death in less time, PZQ-SLN decrease the cytotoxicity in HepG2 cells	de Souza et al. (2014)
2	Liposomal-Praziquantel (Lip-PZQ 300 mg/kg	*S. mansoni*	Mice	Liposome-encapsulated PZQ at 300 mg/kg, is more effective than an equal dose of free PZQ with respect for schistosomicidal potential, the inhibition of worm oviposition and the formation of hepatic granulomata. Lip. PZQ decreases worm count up to 68.8%, the amount in the intestinal and 79% in the liver by and 98.4% in hepatic granulomas as compared to control group free PZQ.	Frezza et al. (2013)
3	MFS-LNC-CTAB^+^ MFS-LNC-OA (20 mg/kg)	*S. mansoni*	Mice	MFS-LNCs offer potential as an alternative single oral dose nanomedicine with a wide therapeutic profile for mass chemotherapy of *S. mansoni*. The number of worms retrieved from the liver and Porto mesenteric vesicles is considerably reduced by both MFS-LNP formulations. The combination of MSF-LNC-CTAB+ was more effective against the young form of worm (91.6 vs. 82.7% drop in worm load respectively). Although the oleic acid (MSF-LNC-OA) formulation was demonstrated to be much more efficient against the immunological early stage than the invading form (96.7% vs. 76.8% drop in worm burden, respectively). This could promote MFS-LNCs as a wider therapeutic profile alternative to PZQ.	El-Moslemany et al. (2016)
4	Miltefosine-Lipid nanocapsules (MFS-LNCs) 20 mg/kg	*S. mansoni*	Mice	MFS-LNC formulation reduces mean worm load to varying degrees (42.31–88.46%). MFS-LNC-CTAB+ and MSF-LNC-OA both led to a larger decrease (MSF-LNC lowers granuloma size by 17.1–31.4%). MFS-LNC-CTAB+ (31.36%) and MFS-LNC-OA (32.99%) showed the greatest reduction. Clinical data suggest that MFS-LNCs could be used as a single-dose oral nanomedicine for *S. mansoni* enhanced therapy instead of praziquantel chemotherapy	Eissa et al. (2015)
5	Praziquantel-Lipid nanocapsules (PZQ-LNCs) (250 mg/kg)	*S. mansoni*	Mice	Encapsulation of PZQ in LNCs resulted in a significant increase in antischistosomal activity upon administration of a single 250 mg/kg oral dose of LNCs, compared to only PZQ suspension to *S. mansoni*-infected mice. Efficacy assessment was based on changes in worm burden count, the size and number of granuloma, and the histopathology of liver Sections. This shows that LNCs as nanocarriers for improving the efficacy of orally administered drugs	Amara et al. (2018)
6	PZQ-Liposomes (8.6 mmol/L)	*S. mansoni*	Mice	In *In-vivo* test PZQ-Liposomes caused a decrease in amounts of eggs and parasites as compared to free PZQ. Liposomes increase the antischistosomal property of praziquantel. While in *in-vitro* study the effect of Liposome_PZQ and free free PZQ were found similar against *S. mansoni* culture	Mourao et al. (2005)
7	PZQ-Liposome + HBO (100 mg/kg)	*S. mansoni*	Mice	When compared to other treatment groups like PZQ, 100 mg/kg of the lip.PZQ + HBO was considered to be more efficient (48% drop of worms, 83.3% drop of egg/Gram of feces) and 100% of mice displayed altered programs (indicating halt of oviposition) compared to other treatments and to the Control group (infected and untreated). The drug were found more available in the body when incorporated into liposomes and used with HBO, the HBO work as an adjuvant	Frezza et al. (2015)
8	PZQ-Liposome 2 mg/mouse	*S. mansoni*	Mice	The encapsulation of PZQ in a liposome can take it to site specific delivery of the drug towards the liver as well as ensuring sustained release properties. The percentage of mice surviving 8 weeks after praziquantel liposome administration exceeds 90% compared to free drug (50%). On the other hand, the number of mice surviving after 14 weeks of administration of praziquantel liposomes is 4-fold greater than the corresponding number of mice surviving after administration of free drug or drug-free liposomes. Along with survival rate Liposome-PZQ has markedly decrease hepatic worm count as compared to free PZQ.	Ammar et al. (1994)
9	Tartar- emetic Liposome 25 mg/kg	*S. mansoni*	Mice	In this study 100% life expectancy was found compared to 28% for the mice injected with drug free liposomes. The liposome-encapsulated drug decrease worm count and shows schistosomal activity significantly	El-Ridy et al. (1989)
10	sulfated polysaccharide α-D-glucan- liposomes (Glu.SO_4_-LIPO) 10 mg/kg	*S. mansoni*	Mice	In this study parasitological analysis revealed that Glu.SO_4_-LIPO was as efficientbas Glu.SO_4_ in reducing egg elimination and worm burden. The use of Glu.SO4-LIPO resulted in a statistically significant decrease in the number of granulomas as free Glu.So_4_	Araújo et al. (2011)
11	Solid lipid nanoparticle-praziquantel (SLN-PZQ) 500 mg/kg	*S. mansoni*	Mice	SLN-PZQ had better bioavailability, absorption rate, and antischistosomal action in all treatment groups, with a larger degree of worm reduction in all dosages studied. The ED95 of SLN-PZQ was 5.29 times less than that of M-PZQ, with significantly higher intestinal tissue egg loads and nearly complete removal of immature implanted eggs in all groups tested	Radwan et al. (2019)
12	Liposome-Praziquantel (Lip.PZQ) 500 mg/kg	*S. mansoni*	Mice	Liposome-encapsulated PZQ resulted in the greatest significant reduction in overall worm amount, egg/Gram in hepatic tissue, and intestine (97.2%, 99.3%, and 99.5% respectively). Lip.PZQ more effective than free PZQ in all aspects, particularly when delivered at 45 days Post-infection. Liposomes can increase bioavailability of drugs in hosts and better absorption by the tegument of *S. mansoni*	Labib El Gendy et al. (2019)
13	Silica-Praziquantel (PZQ-Si) 250 mg/kg	*S. mansoni*	Mice	Mesoporous silica NP is a non-toxic nano-carrier for PZQ, demonstrating anti-schistosomal, antioxidant, immunomodulatory, and anti-inflammatory acts in *S. mansoni*-infested mice. PZQ-Si at a lower PZQ dosage may be recommended for successful PZQ antischistosomal mass chemotherapy. Hepatic DNA fragmentation, measured by comet assay, was significantly improved in infected mice treated with maximium dose of PZQ-Si as compared to positive or PZQ control groups	Tawfeek et al. (2019)
14	Epiisopoturine loaded liposome 300 µg/ml	*S. mansoni*	Hamster	Epiisopiloturine loaded liposome shows 100% mortality of mature parasites in *In-vitro* study after incubation of 96h and 120 h respectively. The worms observed through a confocal microscope give alteration in the morphology and tegument of an adult worm of *S. mansoni*	Guimaraes et al. (2014)
15	Praziquantel-nanostructured lipid nanocarrier 2 and Nanostructured lipid carrier 4 (PZQ-NLC2 and NLC4) 25 µg/ml	*S. mansoni*	Rat	PZQ encapsulated in NLC2 and NLC4 enhances the drug’s safety, efficacy, and therapeutic efficacy against the BH strain of *S. mansoni*. PZQ-NLC2 and PZQ-NLC4 had higher efficacy than free PZQ. The absorption capacity of PZQ NLC4 was discovered to be higher than that of PZQ alone. The intestinal transport of free PZQ and PZQ-NLC2 was similar. However, it is observed that the concentration of PZQ absorbed was smaller when PZQ was loaded in NLC4. The difference between the amounts of absorbed PZQ could indicate that the presence of T60 in the nanoparticles (NLC4) increased the rigid lipid matrix, prolonging release of the drug	Kolenyak-Santos et al. (2015)
16	PZQ-niosomes 250 mg/kg	*S. mansoni*	Mice	The *in-vitro* investigation found that Niosomes combined with PZQ at a concentration of 0.001 g/ml better the death ratio from 30 to 50%, whereas noisome alone exhibited 30% mortality and PZQ showed 10% fatalities. In an *in-vivo* investigation, noisome-PZQ significantly decreased adult worm count, intestinal and hepatic egg deposition, liver granuloma size, and vascular endothelial growth factor expression as compared to free PZQ. This study shows that niosomes as promising carriers for enhanced activity of PZQ.	Zoghroban et al. (2019)
17	PZQ-MFS Lipid nanocapsules (250 mg PZQ + 20 mg miltefosine/kg)	*S. mansoni*	Mice	PZQ-MFS lipid nanocapsule at a dose rate of 250 mg PZQ-20 mg miltefosine/kg demonstrates a substantial increase in antischistosomal efficacy in terms of statistical augmentation in mean worm load, particularly against invasive and early-stage worms, as well as improvement in hepatic granulomas	Eissa et al. (2020b)
18	Liposome Encapsulated oxamniquine	*S. mansoni*	Mice	The liposome encapsulation has prolong the chemoprophylatic effect of oxamniquine. Liposome encapsulated oxamniquine show higer percentage of worm count reduction than free oxamniquine. T-cell and B-cell responsiveness to soluble adolescent Soluble proteins demonstrate that oxamniquine in liposomes activates the immune system of mice against the *S. mansoni* worm	El Ridy et al. (1997)
19	Liposomes Entrapped Oxamniquine (LOXA) 10 mg/kg	*S. mansoni*	Mice	LOXA produced a prominent decrease in the worm load in comparison to other preparations. LOXA shows a maximum reduction (97%) in parasite numbers when LOXA was given s/c 1 day before infection. The results indicate that LOXA is more potent than free OXA when administered through the s/c route at a time close to the infection	Frézard and de Melo, (1997)
20	Praziquantel-miltefosine-nanocapsules PZQ-MFS-LNCs PZQ 250 mg/kg-MFS 20 mg/kg	*S. mansoni*	Mice	When compared to an infected non-treated control, administration of the nano combination one or 7 days before infection resulted in a significantly substantial drop in mean worm burden and granuloma size, as well as improvement in hepatic pathology. It could be an excellent alternative to PZQ in MDA programs by offering the potentials of radical cure due to multistage therapeutic profile, prevention of re-infection because of prophylactic activity, delaying development of resistance to the component drugs in addition to improving patient compliance	Eissa et al. (2020a)

### Nanoparticles (NPs)

Drugs can be administered *via* enteral or parental routes using NPs delivery systems (Tawfeek et al., 2019). NPs have potent antiparasitic effects by acting as a drug carrier for commonly used drugs, such as praziquantel in the treatment of schistosomiasis (Kolenyak-Santos et al., 2015). SLNs-loaded PZQ were found to enhance PZQ’s pharmacological and safety characteristics *in vitro* on *S. mansoni* cultures (Kolenyak-Santos et al., 2015). To treat schistosomiasis, malaria, viserial leishmaniasis (VL), and visceral larva migrans, metal NPs, chitosan (CS), and liposomes are conjugated with a range of medications, including praziquantel, chloroquine, amphotericin B, rifampicin, and albendazole. NPs have the ability to be employed as a vaccine candidate for toxoplasmosis and malaria, as an additive to boost immune response against schistosomiasis, VL, and Chagas disease, and as an additive to increase immunological response against schistosomiasis, Visceral leishmaniasis (VL), and Chagas disease (Abaza, 2016). Gold nanorods were combined with a recombinant *S. mansoni* tegument protein Sm29 to immunise mice against schistosomiasis (Assis et al., 2018). AuNPs are being used to alleviate oxidative stress in mice spleen tissue produced by *S. mansoni* infection (Dkhil et al., 2017). Other nanoparticles, such as liposome nanoparticles, were also used *in vitro* to transport epiisopiloturine, with promising results (Tawfeek et al., 2019), as well as poly (lactic-co-glycolic acid) (PLGA) nanoparticles loaded with lignan (-)-6,6′-dinitrohinokinin (DNHK) for schistosomiasis treatment (Tawfeek et al., 2019).

**FIGURE 1:**
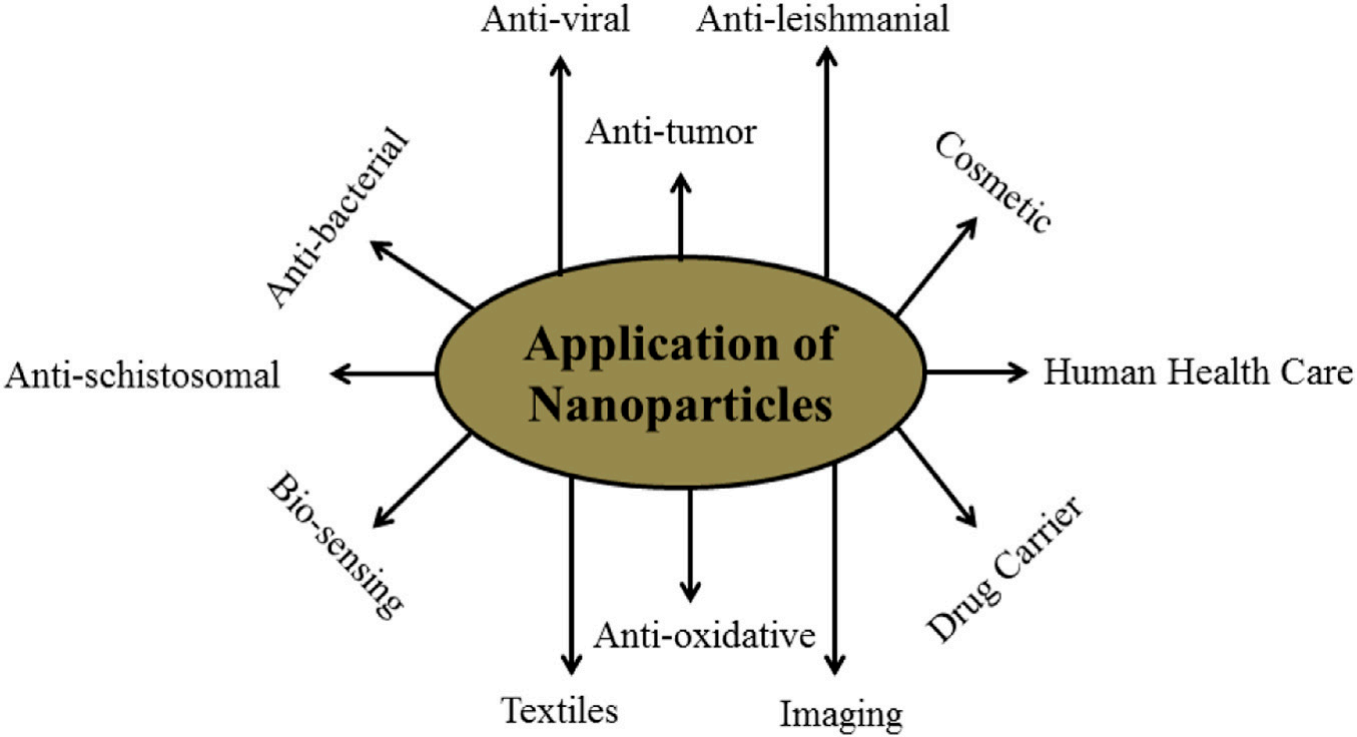
Schematic diagram of applications of Nanoparticles in various fields.

**FIGURE 2:**
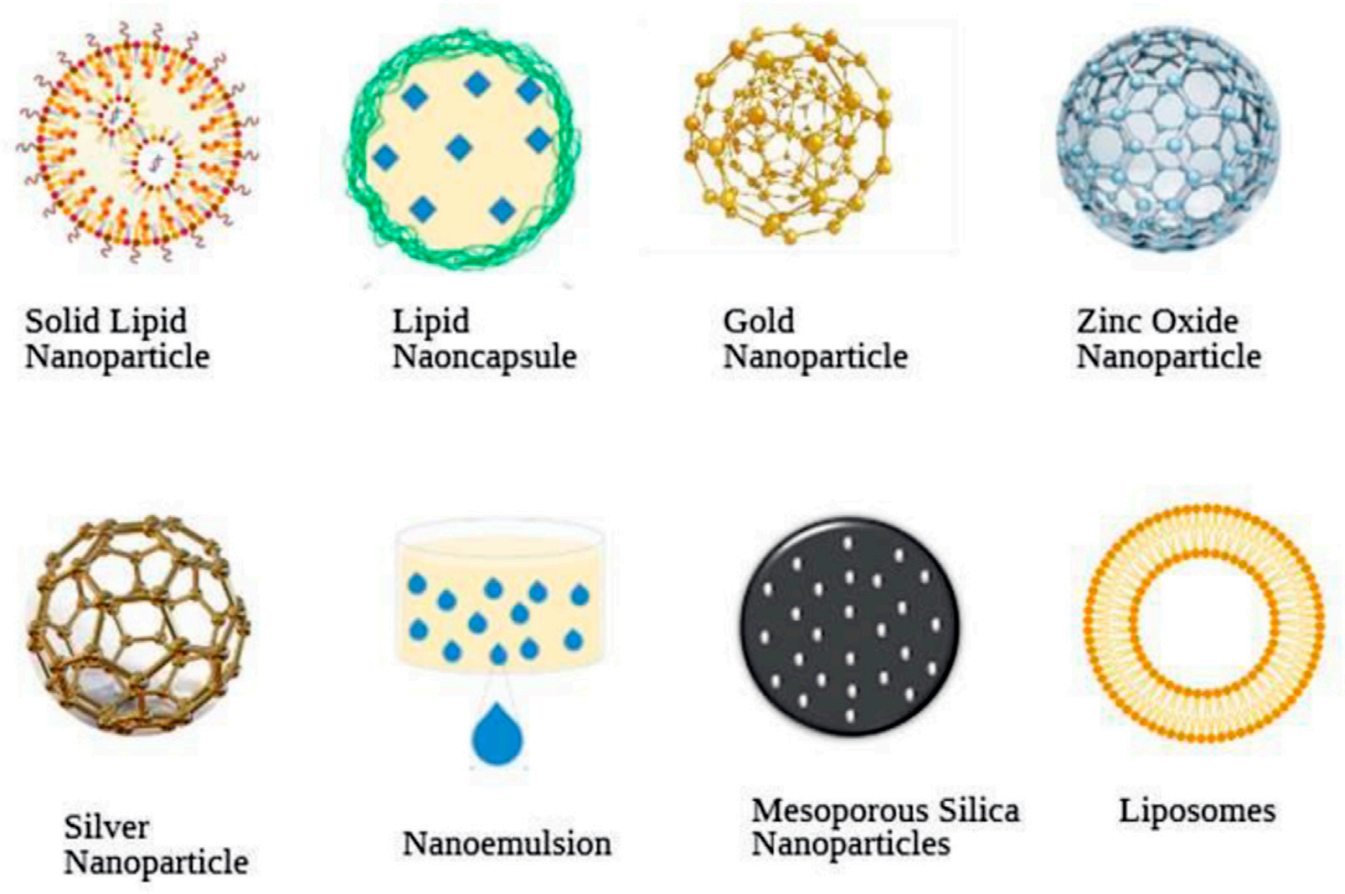
Schematic diagram of various commonly used nanoparticles in schistosomiasis treatment, diagnosis, and prevention.

Nanoparticles are used in various types of products for different type’s activities. Nanoparticles uses in various fields in given in (Figure 1). Based on shape, size, and chemical characteristics, NPs are commonly classified into different categories. Some of the well-known NPs used in drug delivery, diagnosis, and vaccine are shown in (Figure 2) and each one is discussed below in detail.

### Gold nanoparticles (AuNPs)

AuNPs have recently been actively used in several fields of nanomedicine for diagnostic and therapeutic purposes. Due to the promising properties of AuNPs, they can be used in near future for medical purposes in almost all fields (Dykman and Khlebtsov, 2011). The gold complexes have also shown possible antileishmanial and antimalarial action (Vieites et al., 2009). AuNPs also show anthelminthic efficacy as investigated in the *invivo* study (Kar et al., 2014). AuNPs were discovered to cause cestode paralysis and death, which the scientists ascribe to alterations in the parasite’s enzymatic activity (Tikariha et al., 2012). Consequently, the use of Au is significant for the treatment of tropical human diseases (de Almeida and Carabineiro, 2013). For example, when AuNPs are used in a polymerase chain reaction (PCR), it increases and improves the specificity of this diagnostic technique (Li and Ji, 2015). In schistosomiasis AuNPs inoculated intraperitoneally can reduce the total worm load, eggs in the liver, and granuloma size in *S. mansoni* infected mice. In addition, the treatment can decrease liver damage due to its anti-inflammatory and antioxidant properties (Dkhil et al., 2015a). Another study discovered that AuNps have a unique therapeutic potential against renal diseases caused by *S. mansoni* infection. AuNPs decreased oxidative stress and histological damage in the kidney. They also restored the expression of the damaged kidney genes (Dkhil M. A. et al., 2016b). Meanwhile, other researchers are employing AuNPs as a means of delivering nuclear-oriented drugs into biological cells (Gu et al., 2009).

### Silver nanoparticles (AgNPs)

AgNPs are amongst several metallic NPs which are commonly used in a range of biomedical applications owing to their fascinating antimicrobial and anticancer properties. In the field of nanoscience and nanotechnology, especially in nanomedicine AgNPs plays a pivotal part in the treatment of various disease (Zhang et al., 2016). The biological activity of AgNPs is determined by factors such as surface chemistry, mass, shape, atom morphology, coating/capping, agglomeration, dissolving rate, and atom reactivity in solution. The efficacy of ion discharge capacity, cell types, and the kind of reducing agent employed in the production of AgNPs are all important factors in determining cytotoxicity (Carlson et al., 2008). AgNPs’ exploitation has attracted considerable courtesy and effort in numerous fields. Though, the antibacterial actions of the new silver type are still largely at the center of the healthcare arena. AgNPs have been widely used in commercialized products and entered into clinical practice, taking benefit of the sterilizer and antibacterial properties of AgNPs (Lansdown, 2006; Cohen et al., 2007). Few studies have provided detail on AgNP’s (silver ions Ag^+^), showing anti-schistosomal activity (King and Highashi, 1992; Cheng et al., 2013). It was discovered that Ag+ was toxic to *S. mansoni* cercariae at concentrations more than 0.09 mM, but that Ag+ was non-toxic at lower levels yet hindered their penetration into linolenic acid-impregnated agar (King and Highashi, 1992). Another study evaluated the impact of AgNPs on schistosome cercariae and its potential use in schistosomiasis prevention, specifically in terms of pathogen transmission. Ag + may contribute largely to the effects of AgNPs on schistosome cercariae. Yet, the possible contribution of nano dimensions of AgNPs could not be ruled out, as remarkably unusual physicochemical properties and biological activities of various nano-sized materials including AgNPs have been demonstrated (Cheng et al., 2013). Owing to the traditional adverse properties of chemotherapy and radiation therapy, scientists and the industry have investigated the idea of utilizing AgNPs as a therapeutic and anti-cancer agent (Zhang et al., 2016).

### Solid lipid nanoparticles (SLNs)

SLNs are solid colloidal elements that are available in many sizes ranging from 30–1,000 nm (Ayan et al., 2017; Ganesan et al., 2018). SLN is made up of solid lipids (lipids at room and body temperatures) that have been stabilized by surfactants. The lipids could be purified triglycerides, complicated glyceride combinations, or even waxes (Wissing et al., 2004). SLNs are encapsulated or embedded in lipid nuclei to form a stable colloidal DDS, and the carriers are usually natural or synthetic solid lipid materials such as saturated fatty acid glycerides, stearic acid, beeswax, cholesterol, cetyl palmitate, monostearate mononitrate, and solid paraffin (Tapeinos et al., 2017; Mishra et al., 2018). High compression homogenization, a microemulsion method, film-ultrasonic diffusion, an emulsification-solvent evaporation method, and hot melt ultrasound technology may all be used to produce SLN (Sohail et al., 2020).

SLNs have extensive application in the delivery of Phyto-bioactive compounds for the treatment of various chronic infections. Because of their outstanding biocompatibility and biodegradability, SLNs have been demonstrated to be a viable alternative to drug delivery methods (Qi et al., 2012; Lasoń et al., 2013). The several benefits of SLNs delivery contain bypassing 120–200 nm particle size in the spleen or liver filtration, improved stability, leakage stoppage, and increase lymphatic endorsement of the bioactive compound through oral delivery (Ayan et al., 2017; Ganesan et al., 2018). SLNs, on the other hand, have drawbacks such as restricted drug-loading capacity and drug ejection due to crystallization during loading circumstances (Rajabi and Mousa, 2016). SLNs can be made from triglycerides, complex glycerides, and waxes (Mader, 2006). Because of their tiny size, SLNs may be used in several modes, including parenteral, pulmonary, and cutaneous applications, as well as an intravenous injection (Üner and Yener, 2007). SLNs have been widely studied for drug administration of dynamic anti-cancer complexes, improving medication oral bioavailability, protecting labile anti-cancer medicines, and reducing adverse effects by selectively targeting the active site (Pindiprolu et al., 2019).

SLN enhanced PZQ’s antischistosomal action, resulting in significantly prolonged drug release due to the encapsulated drug’s protection from enzymatic destruction. Despite the prolonged residency in the systemic circulation, oral administration of this SLN-PZQ demonstrated a substantial improvement in bioavailability and effectiveness with a safer profile when compared to traditional medication (Radwan et al., 2019). Another study found that PZQ-loaded SLN might be a new drug delivery method for schistosomiasis therapy, particularly in marginalized communities, improving therapeutic effectiveness while reducing PZQ side effects (de Souza et al., 2014). The use of hydrogenated castor oil in SLN increased PZQ bioavailability as well as drug residence duration after oral delivery (Xie et al., 2010). PZQ-loaded SLN was used to investigate the biological uses of this technology for PZQ intestinal penetration (de Souza et al., 2014).

## Lipid nanocapsules (LNCs)

LNCs feature a hybrid shape between polymeric nanocapsules and liposomes due to their oily core enclosed by a rigid capsule (Huynh et al., 2009). Miltefosine, a medication used to treat cutaneous breast cancer metastases and Visceral leishmaniasis, was shown to have improved availability and activity due to improved gastrointestinal transit and schistosomicidal activity when encapsulated in LNCs and taken orally, according to one study (Eissa et al., 2015). Due to the schistosomes’ better-absorbed tegument, which has an attraction for the phospholipid bilayer, the usage of LNCs has recently received significant attention, notably in the treatment of schistosomiasis. Because of their amphipathic character, LNCs can play a prominent part in modifying the solubility and rate at which drugs such as PZQ can be targeted, therefore increasing penetration across biological barriers (Cheng et al., 2017).

Moreover, a single dose of PZQ-LNCs substantially improve efficacy and decreased worm burden, hepatic pathology improvement, and substantial damage to the fluke suckers and tegument (Amara et al., 2018). Whereas PZQ-lipid Nano-capsule at a dose rate of 250 mg PZQ-20 mg miltefosine/kg demonstrates a substantial increase in antischistosomal efficacy in terms of statistical augmentation in mean worm load, particularly against invasive and early-stage worms, as well as improvement in hepatic granulomas (Eissa et al., 2020b), The prophylactic role of a single oral dose of PZQ-MFS LNCs was evaluated and demonstrate that when compared to an infected non-treated control, administration of the nano combination one or 7 days before infection resulted in a significantly substantial drop in mean worm burden and granuloma size, as well as improvement in hepatic pathology (Eissa et al., 2020a).

Likewise in one more investigation, the miltefosine encapsulation method was employed in LNCs remodeled with cetyltrimethylammonium bromide (CTAB), a +ve charge conveyance substance, or oleic acid with dose directed orally in *S. mansoni* infected mice. According to the findings of this investigation, a single oral dose of both encapsulated miltefosine resulted in a drop in parasite load as well as a reduction in liver granulomas in infected mice. Following the previous study findings, the investigators found that the role of encapsulated miltefosine is a feasible nanomedicine for mass schistosomiasis chemotherapy, particularly its application as an oral single dosage (El-Moslemany et al., 2016).

### Zinc oxide nanoparticles (ZnO-NPs)

ZnO-NPs are employed in a variety of industrial products such as rubber, dye, coating, and cosmetics. ZnO-NPs is one of the most extensively utilized metal oxide NPs in biological applications due to their excellent biocompatibility, low toxicity, and low cost. ZnO-NPs have emerged as potentially useful in biomedicine, particularly in the anticancer and antibacterial domains, due to their ability to trigger the creation of extra reactive oxygen species (ROS), the release of zinc ions, and the induction of apoptosis (Jiang et al., 2018). Dietary ZnO-NPs improved oxidative status and improved the efficiency of several blood enzymes. As a result, ZnO-NPs were employed as a novel *leishmania* therapy (Ahmadi et al., 2014; Nadhman et al., 2014), and schistosomiasis (Bauomy, 2020). The photo-activated disinfectant effectiveness of the Ag@Sm-doped ZnO/CB nanocomposite for *Staphylococcus aureus* (80%), *Pseudomonas. Aeruginosa* (60%), and *S. mansoni* cercariae (100%) was associated with gradual degradation in the cercarial body. This nanocomposite is also effective for adult *S. mansoni* worms, leading to near-complete worm mortality and substantial worm body destruction. These findings validated Ag@Sm-doped ZnO/CB as a powerful biocide weapon capable of destroying harmful bacteria and parasites in both dark and light settings (Darwish et al., 2018). Another study revealed that administering ZnO NPs and/or L-carnitine to schistosome-infested mice decreased brain oxidative stress measures, with glutathione levels and catalase activity much greater than in the schistosome-infested group. On the contrary, the therapy dramatically reduced the levels of nitrite/nitrate, malondialdehyde, and reactive oxygen species. Furthermore, the treatment with ZnO nanoparticles and/or L-carnitine cured the neuro-schistosomiasis-related brain histological abnormalities (Bauomy, 2020).

### Liposomes

Liposomes are small structures used as a drug delivery system to reach tissues, delivering only a portion of the drug to the target site (Tomiotto-Pellissier et al., 2017). Liposomes are amphiphilic, meaning they can transport both hydrophobic and hydrophilic medicines. It can protect the encapsulated medications by isolating them from the outer environment, increasing the solubility of the lipophilic drug, and extending the action period of the drugs (Muthu et al., 2011). Liposomes’ physiochemical characteristics may be changed, permitting the encapsulated drug to be delivered to the exact targets, resulting in greater therapeutic efficiency than traditional dose forms (Ammar et al., 1994). Because of their great biocompatibility and low toxicity, liposomes have sparked a lot of interest as a drug delivery system (Mufamadi et al., 2011).

In the treatment of *S. mansoni*, praziquantel (300 mg/kg) encapsulated in liposomes significantly reduced worm load, stool and intestinal egg count, and the number of hepatic granulomas (Frezza et al., 2013). In *S. mansoni* infected mice sulfated polysaccharide -D-glucan extracted from *Ramalina celastri* encapsulated in liposomes significantly reduced hepatic granuloma (Frezza et al., 2015). Thus, liposomes have the benefit of not restricting the drug’s physicochemical characteristics that are not enclosed, such as hydrophilicity or membrane, improving the therapeutic efficacy, regulating intake and tissue distribution, and producing less toxicological effects that encourage schistosomiasis treatment (Tomiotto-Pellissier et al., 2017).

### Mesoporous silica nanoparticles (MSNs)

MSNs are utilized as a nano-drug carrier because of their unique organization and remarkable features such as higher specific surface area (>900 m^2^/g^−1^) and lower volume (>1 cm3/g-1), ordered apertures, variable pore diameter (2–50 nm), excellent biocompatibility, and thermal stability (Li et al., 2017). These consistent surface characteristics and structures are ideal for encapsulating proteins, microbes, and other sorts of drugs. Furthermore, MSNs’ mesoporous characteristics allow them to carry a high amount of anticancer drugs and enable drug assortment *via* nano-sized components *via* passive targeting to the tumor location. As a result, MSNs are employed as an effective drug delivery system for a variety of diseases (Sohail et al., 2020). Furthermore, suitable surface functionalization on the external surface of MSNs can be performed to get a dynamic targeting mechanism. Such as chemicals sensitive to exterior stimuli like as pH, magnetic field, temperature, redox, and enzymes are utilized to manufacture environmentally friendly and controllable DDS to improve anticancer medication targeting and diminish side effects (Pan et al., 2012). Considering MSNPs’ potential to increase PZQ bioavailability and shorten half-life in the exchange (Amara et al., 2018). PZQ-Si has superior physiochemical properties such as tiny constant size (105 nm), spherical shape, and PZQ trapping efficacy (83%). In terms of overall worm load, tissue egg count, oogram pattern, and liver granuloma count and diameter, the PZQ-Si therapy produces the best antischistosomal results when taken orally. According to the findings, mesoporous silica NP is a likely safe nanocarrier for PZQ, enhancing its antischistome, antioxidant, immunomodulatory, and anti-inflammatory action in *S. mansoni*-infected mice (Tawfeek et al., 2019). Silica nanoparticles were effectively produced and analyzed, showing acceptable features for *in vivo* uses. Soluble worm antigenic preparation (SWAP) has been included in the MSNs. SWAP-loaded MSNs outperformed a conventional vaccination system in terms of immunization performance (SWAP-associated aluminum salt). These findings imply that SWAP-loaded MSNs is a potential approach for improving the immune response to *S. mansoni* (de Pádua Oliveira et al., 2016). MSNs have revolutionized nanobiotechnology due to their ability to contain a high number of drug molecules. In comparison to crude drug material, they were extremely successful in the delivery of hydrophobic drugs, considerably improving their solubility and bioavailability after oral administration (Zhang et al., 2012). Increasing the performance of already existing medicines is thought to be a more beneficial strategy from an economic standpoint (Tomiotto-Pellissier et al., 2017).

### Nanoemulsions

Nanoemulsions are tiny biphasic particles where it is one component is firmly dispersed into the next in the shape of microscopic droplets ranging in diameter from 10–600 nm and are used to transport oil-soluble substances (de Araújo et al., 2007). It can be an oil-in-water nanoemulsion or a bi-continuous nanoemulsion with oil and water interspersed throughout the system (Tomiotto-Pellissier et al., 2017).

Although aminoalkanethiosulfuric acids are a class of chemicals with schistosomicidal action, their use as a pharmaceutical preparation is restricted due to their low water solubility (de Oliveira Penido et al., 2008). The efficacy of 2-(butyl amino)-1-phenyl-1-ethane thiosulphuric acid (BphEA) utilizing nanoemulsions incorporating BphEA is enhanced. This nanoemulsion was capable of inducing worm motor activity loss, tegument disruption, female worm death, and male worm sluggish movement. When the parasites were treated with a free compound, these outcomes were not observed (de Araújo et al., 2007).

### Nanotechnology applications in the field of nanomedicine

**FIGURE 3:**
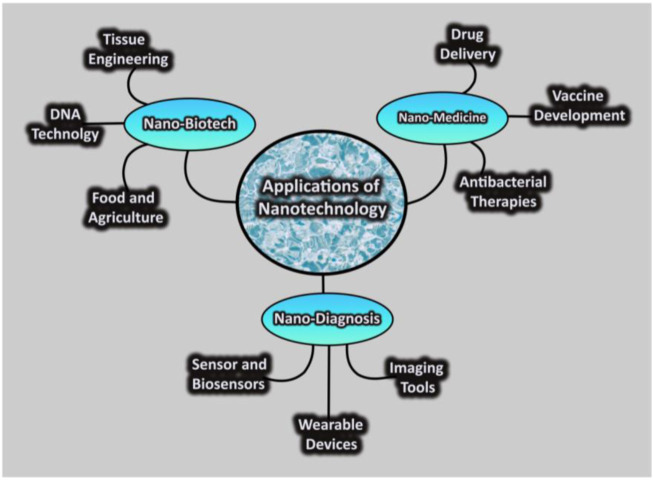
Schematic diagram of nanotechnology application in various fields.

In the field of nanomedicine, the use of nanotechnology aims to improve public health and quality of life (Jia, 2005). Currently, nanotechnologies are used in various fields such as nano-medicine, nano-biotechnology, and nano-diagnosis as shown in (Figure 3).

### Nanomaterial as therapeutic agents

**FIGURE 4:**
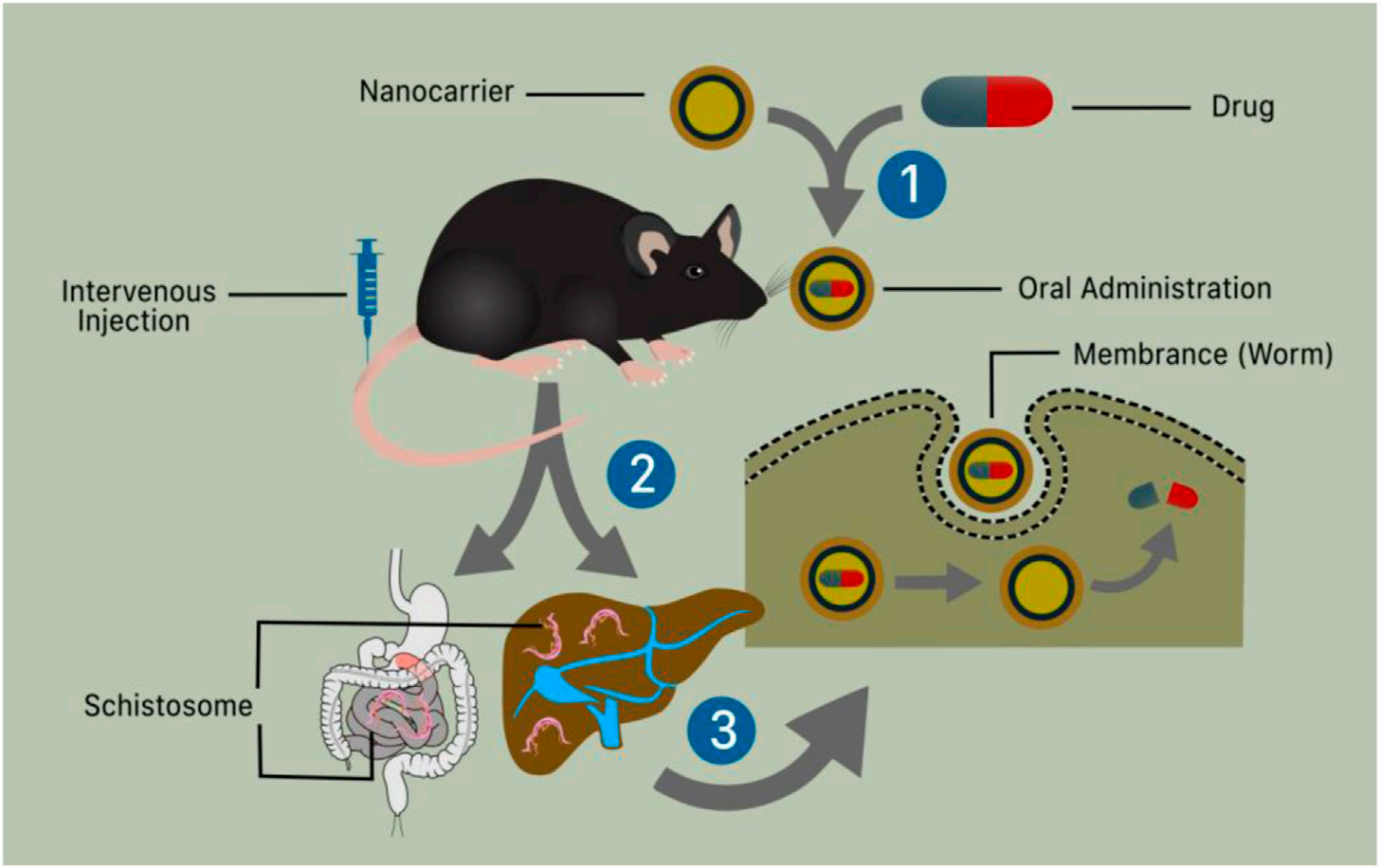
Schematic illustration of nanotechnology and nano-enable drug delivery system use in schistosomiasis treatment. (1) The liver and digestive system containing schistosomes that enter the body by skin penetration and reach the specified organ through the bloodstream (2) The oral and intravenous administration of a nanotechnologically based medication causes the worms’ membrane (tegument) to rupture, permitting the drug to be distributed and kill the worms.

Nanomaterials have been industrialized for their usage in medicine and several types of gadgets. Because of their antimicrobial properties, NPs of certain metals or metal oxides are now broadly used to treat various disorders and improve human health. NPs with particular physicochemical properties have demonstrated antimicrobial, antiviral, and antiparasitic properties (Jebali and Kazemi, 2013). Because of their small particle size and charged surface, nanoparticles can easily enter pathogenic cells and interfere with cellular contents such as protein and DNA, inducing programmed cell death (Sharmin et al., 2021). Antileishmanial capabilities are exhibited by gold and silver nanoparticles, as well as zinc, titanium, and magnesium oxide nanoparticles (Jebali and Kazemi, 2013). Furthermore, gold nanoparticles (GNPs) exhibited antischistosomal activity in the liver and brain of mice (Dkhil et al., 2015a). Treatment is a substantial therapeutic action. While it is favored that prevention of diseases is better than treatment, therapy for an already present disorder is necessary (Emerich, 2005). The main objective of treatment is to make the patient as comfortable as possible. For millennia, therapy has been evolving at a rapid pace. The introduction of Nano therapy healthcare has given rise to a new promise in medicine (Moghimi et al., 2005; Murthy, 2007). The application of nanomaterial as a new addition to enhance the conventional therapeutic procedure can be very useful (Nakayama and Okano, 2005; Gulati and Gupta, 2012). Nanomaterials and the use of a nano-enabled drug delivery system (Figure 4) have been a key emphasis with the hope of enhancing treatment results for schistosomiasis using PZQ. Table 2 lists the various nano-therapeutic compounds utilized as anti-schistosomal therapy for the treatment of diseases**.**

**TABLE 2:** List of various nanoparticles used therapeutically in the treatment of schistosomal infections.

S. No	Nanoparticles used	Schistosomes species studied	Model animal used in the study	Efficacy	References
1	Gold nanoparticles (AuNPs) 0.25, 0.5, and 1 mg/kg	*S. mansoni*	Mice	In comparison to the control group, AuNPs dramatically reduce overall worm load, egg load in liver granuloma size, malondialdehyde activate and nitric oxide levels, and enhance glutathione levels. This also decreases mRNA expression of interleukin-1, interleukin-6, tumor necrosis factor, interferon, and inducible nitric oxide syntheses. This result indicates that Au-NPs are being effective anti-schistosomal and antioxidant agents. AuNPs used at a higher dose 1 mg/kg were found more effective than free PZQ in worm burden and egg count	Dkhil et al. (2015a)
2	Gold Nanoparticle (AuNPs) 1 mg/kg b.wt	*S. mansoni*	Mice	Au-NPs therapy considerably lowers splenic levels of nitrite/nitrate and MDA in splenic tissue as a consequence of Au-NPs and PZQ treatment; NPs also enhance the level of GSH in infected mice gut and spleen. Au-NPs significantly improve histological images of the spleen with specific histological impairments than the non-infected control group	Dkhil et al. (2017)
3	Gold Nanoparticles (AuNPs) 250,500 and 1,000 µg/kg	*S. mansoni*	Mice	AuNPs significantly reduce the level of nitrite/nitrate and MDA, AuNPs at a dose rate of 250 µg/kg showed non-significant changes in KIM-1 and significant up-regulation of NGAL mRNA expression, whereas MCP-1 and TGF-β was an expression of mRNA which significantly reduced. AuNPs treatment in mice reduced the extent of histological impairment and renal oxidative damage. AuNPs were able to regulate gene expression impaired by *S. mansoni* infection	Dkhil et al. (2016b)
4	Selenium nanoparticles (Se-NPs) (0.5 mg/kg)	*S. mansoni*	Mice	Injection of selenium nanoparticles into *Schistosoma* infected mice enhanced hepatic histopathology and reduced the diameter of granulomas. The treatment increased the level of glutathione. While the nitrite/nitrate and malondialdehyde levels decrease significantly. The result suggested that the Se-NPs in infected mice with *S. mansoni* work as an Anti-schistosomal drug	Dkhil et al. (2016a)
5	Zinc oxide Nanoparticles (ZnO-NPs) 5.6 mg/kg	*S. mansoni*	Mice	In schistosome-infected mice, treatment with ZnO-NPs declined brain oxidative stress parameters, with glutathione and catalase activity considerably enhanced. The therapy, also significantly decreased the levels of nitrite/nitrate, malondialdehyde, and reactive oxygen. ZnO-NPs treatment also improved the neuro-schistosomiasis-related brain histopathological impairment and restored the DNA laddering profile	Bauomy, (2020)
6	Gold nanoparticles (Au-NPs) 1 mg/kg Selenium nanoparticles (Se-NPs) 0.5 mg/kg	*S. mansoni*	Mice	To test the effect of the nanoparticles, infected mice were fed them individually. The parasites caused a substantial reduction in glutathione levels after injection; nevertheless, the levels of nitric oxide and malondialdehyde were greatly augmented. The treatment of mice with metal nanoparticles dramatically reduced the levels body weight changes, oxidative stress, and histological alterations in the jejunal tissue significantly. Thus they have proved their potential anti-schistosomal activities in mice successfully	Dkhil et al. (2019)
7	Silver Nanoparticles (Ag NPs) 125 µg ml^−1^	*S. japonicum*	Mice	In a dose-dependent fashion, AgNPs produced cercarial tail-shedding, disturbed behavior, and a reduction in cercarial secretion. It was discovered that extended treatment was cercariocidal, which might be attributed to AgNP-induced cercarial tail loss rather than toxicity. Ag + may be important in the effects of AgNPs on schistosome cercariae. Non-etheless, given the remarkably unusual physicochemical properties and biological activities of various nano-sized materials, including AgNPs, the possible contribution of AgNPs’ nano dimensions could not be ruled out	Cheng et al. (2013)
8	Au-NPS (0.25, 0.5, and 1.0 mg/kg)	*S. mansoni*	Mice	Au-NPS could reduce the neurooxidative stress and control the gene expression in the brain of diseased mice. The results show that GNPs have an antischistosomal effect against *S. mansoni*. The neuro-schistosomiasis treatment with GNPs improved the histopathological changes. A significant decrease (*p* ≤ 0.05) was noticed in DA content in schistosome-infected mice treated with 0.25 and 0.5 mg/kg GNPs and+PZQ as compared to the non-infected control group. On contrary, the administration of the higher dose of GNPs (1.0 mg/kg) resulted in a significant elevation in DA content *versus* the control group. The content of brain DA showed a significant increase in all treated groups as compared to the schistosome-infected group	Dkhil et al. (2015b)
9	Cur-GNPs	*S. mansoni*	Mice	Curcumin-loaded gold nanoparticles (Cur-GNPs) combined with PZQ reduce worm load in the third week, with the largest drop in the intestine and liver egg content and a 70.1 decrease in granuloma size. This study showed that curcumin when combined with PZQ, has substantial antischistosomal properties against *S. mansoni* through altering biochemical, histological, and immunological alterations as compared to the untreated control group	Mokbel et al. (2020)
10	Ag-NPs (50 μg/ml) + Au NPs (100 μg/ml)	*S. mansoni*	Mice	In *in-vitro* research, Ag-NPs and Au-NPs were used at dosage rates of 50 g/ml and 100 g/ml, respectively, *S. mansoni* cercariae morality was enhanced in a dosage and time-dependent manner, reaching 100% mortality after 1 h of incubation. In an *in-vivo* trial, it reduced worm load, and egg count/g in the gut, and liver as compared to the control group	Moustafa et al. (2018)
11	TA-Long-circulating pegylated liposomes (LCL) 11 mg Sb/kg	*S. mansoni*	Mice	When related to the control group (untreated or treated with empty LCL), the LCL-treated cluster has a considerable drop in worm load (55%). According to the findings of this investigation, LCLs decrease toxicity and efficiently transport TA into *S. mansoni* in the last stage of parasite infection. The present work demonstrates that LCL reduces the acute toxicity of TA and effectively deliver this drug to *S. mansoni* during the late stages of parasite infection	de Melo et al. (2003)

### Nanotechnology use in schistosomiasis diagnosis

Schistosomiasis diagnosis is essential for the recognition and treatment of the diseases in prevalent and non-prevalent areas, as case finding, morbidity valuation, and control plans are all based on the result from diagnosis (Ajibola et al., 2018; Odundo et al., 2018). Current diagnostic methods for schistosomiasis include traditional parasitological methods, immunological diagnosis, as well as molecular methods (Katz et al., 1972). Common traditional parasitological methods include the use of a microscope for parasitic egg determination in urine and stool, or by immunological method (antibody or antigen detection) (van Etten et al., 1994; Odundo et al., 2018). Kato-Katz method is cheap and convenient and offers a high degree of specificity (Caldeira et al., 2012). The sensitivity of the test is contingent on the degree of the disease, the technique, and the judgment of the post-infection host. The understanding of existing antibody analyses is not ideal (ranges from 65 to 86 percent), (Odundo et al., 2018). Screen printing electrode biosensors have recently attracted significant attention in clinical sciences, food, and drug analysis process control. These sensors can determine very minute amounts of analytes by detecting the changes in potential, current, and conductance caused by an immune response (Taleat et al., 2014). Nanotechnology has been used to enhance the correctness and improve existing methods, but also provide new techniques unexpectedly (Lin et al., 2008; Yang M. et al., 2009b). The use of NPs in immunosensing has demonstrated great potential in the development of highly sensitive, versatile diagnostic care devices (Dequaire et al., 2000; Baptista et al., 2008; Wan et al., 2013). The list of various nanosensors used to boost the diagnostic effectiveness against schistosomal infections is provided in Table 3.

**TABLE 3:** List of Nano-sensor/Nano-material used in improving the diagnostic ability against schistosomal infections.

S. No	Nano-sensor nano-materials used	Schistosomes species studied	Num. of schistosoma positive sample in study	Efficacy	References
1	AuNPs-Mab/Elisa	*S. mansoni*	71	ELISA’s sensitivity and specificity for detecting Circulating Schistosomal antigen (CSA) using AuNPs-Mab was 100% and 97.8%. A more significant positive correlation was detected on the use of AuNPs-Mab/ELISA (*r* = 0.882). Loading AuNPs with Mab (6D/6F) improved the precision of sandwich ELISA for the determination of CSA, allowing active and mild infections to be identified easily	Kame et al. (2016)
2	AuNP-IgG Nano-sensor	*S. mansoni*	……………	Immobilized AuNPs combined with bilharzia antibodies proved their diagnostic potential. The detection range of bilharzia antigen in stool samples was 1.13 × 10^−1^ ng ml^−1^ to 2.3 × 10^3^ ng ml^−1^, with a detection limit of 8.3887 × 10^−2^ ng ml-1, showing the ability of the nano-biosensor for detection of bilharzia antigen in stool samples	Odundo et al. (2018)
3	NCE-AGs	*S. mansoni*	………………	The proposed NCE electrode’s quantitative response and great sensitivity to Abs of *S. mansoni are* as low as 38 pg/indicateting that it may be developed as a site-user, low-cost, and rapid electrochemical immuno-sensor	Shohayeb et al. (2016)
4	AuNP-IgG Conjugate	*S. mansoni*	………………	Conjugate was tested as the analyte with a differing concentration of conjugate soluble Egg Antigen (SEA). The single response was directly proportional to the SEA concentration. A SEA concentration plot against the current change was obtained. The detection limit of 3.31 × 10^−5^ ng/ml was obtained with formula 3σ/slope, where σ is the standard deviation of three blank solutions	Naumih et al. (2016)
5	MBA-Fe3O4-NPs-AuNPs-DNA Probe System	*S. mansoni*	………………	On the changed surface of the electrode, the probe system exhibits an efficient electrochemical response. At varied DNA quantities in the genome, the proposed biosystem was capable of identifying *S. mansoni* unique nucleotide sequences in cerebrospinal fluid (CFS) and blood samples. At higher DNA concentrations, bio recognition caused an increase in electron transfer resistance and a decrease in current peaks during electrochemical testing. The established platform had detection limits of 0.781 and 0.685 pg L-1 DNA for serum and CFS, respectively	Santos et al. (2017)
6	MPTS-AuNPs-DNA Probe System	*S. mansoni*	………………	The proposed biosystem detected the *S. mansoni* genome sequence in urine samples, cerebrospinal fluid system, and serum in varying amounts. It measured concentrations in urine (27–50 pg L^−1^), cerebral fluid (25–60 pg L^−1^), and serum (27–42 pg L^−1^). The limit detection (LOD) of the biosensor was 0.6 pg μL^−1^. The developed labeled free genosensor was able to detect small concentrations of *S. mansoni* DNA in complex biological fluids	Santos et al. (2019)
7	GICA	*S. japonicum*	174	GICA shows a sensitivity of 93.7% in patients with Schistosomiasis and 97.6% specificity in the healtyh population and patients with other parasitic diseases.Partially purified SEA in GICA is effective for detecting schistosomiasis in the fields since it requires a little amount of blood, has high sensitivity, and has a low cross-reaction rate	Shou-fu et al. (2014)
8	GICA	*S. japonicum*	100	GICA identification strips of *S. japonicum* in mice, rabbits, buffaloes, and goats show high sensitivity (100% in each spp.) and specificity (100%, 100%, 94.23%, and 88.64% respectively). When compared with ELISA, the GICA strips exhibited similar sensitivity and specificity in the diagnosis of schistosomiasis in mice, rabbits, buffaloes, and goats. Besides, only 5 μl of serum is required for the test and the detection can be completed within 5 min	Xu et al. (2017)

### Nanotechnology and vaccine production

Vaccination has a significant influence on infectious disease control. There are a lot of serious diseases, yet no effective vaccine is available for them (Gregory et al., 2013). Nanotechnology is playing an increasingly important role in vaccine development. Measures that improve antigen efficiency are becoming increasingly essential as vaccination development focuses on less immunogenic “minimalist” formulations. The use of NPs in vaccine manufacturing increases not only antigen storage and immunogenicity but also permits targeted administration and slow release (Zhao et al., 2014). Nanotechnology usage has grown exponentially in recent decades, giving rise to the term “nanovaccinology” (Mamo and Poland, 2012). Nanoparticles are used in both prevention and treatment. It can be used as a vehicle for antigen delivery or as an immunostimulant adjuvant to activate or boost immunity. Prophylactic nanovaccinology is used to regulate and prevent various illnesses, whilst therapeutic nanovaccinology is used to cure cancer (Bolhassani et al., 2011; Krishnamachari et al., 2011). Traditional vaccinations contain live attenuated microorganisms, dead bacteria, or components of microbes. While many of these vaccinations were essential for controlling infectious organisms, many of them did not give protection against specific diseases (Gregory et al., 2013). A range of infectious diseases lacks licensed vaccines, as most of them are purified proteins, polysaccharides, or naked DNA encoding a protective antigen (Harandi et al., 2010). Control methods for schistosomiasis are primarily grounded on chemotherapy, but given eras of bulk action, the number of people diseased remains persistent (Harder, 2002). General prevalent zones have continuous reinfection of people and poor sanitation situations in the evolving countries, making drug treatment alone inadequate (Bergquist and Colley, 1998). Researchers find that the long period strategy for managing schistosomiasis is through combining immunization with drug treatment (Bergquist, 2002). An antischistosomal vaccine that even partially reduces worm loads might significantly decrease the pathology and limit the infection with a parasite (Chitsulo et al., 2004). The researcher has now redirected the usage of nanotechnology as vaccine delivery vehicle for treatment. The vaccine antigen would either be enclosed or imprinted on the exterior of the NPs. By packaging antigenic components, NPs allows for the administration of antigens that would otherwise be quickly degraded after injection or trigger a localized immune response. The conjugation of antigens to NPs allows the immunogen to be given to the immune system in the same way as the pathogen would, resulting in a comparable reaction (Gregory et al., 2013). Nanotechnology is used because of its greater stability and easier accessibility to the target site. The list of various nanoparticles used along with vaccine materials against schistosomal infections is given in Table 4.

**TABLE 4:** List of various nanoparticles used along with vaccine materials against schistosomal infections.

S. No	Nanosensor used	Schistosomes species studied	Animal model used	Efficacy	References
1	NPs-coated pVR1020-SjGST DNA vaccine	*S. japonicum*	Mice	Positive immunological responses were generated by the NP-coated DNA vaccine formulation. It induces a significantly enhanced immune response, a T-helper 1 polarized cytokine milieu, a higher proportion of IFN-γ producing CD4^+^ T-cells, and a concomitant decrease in IL-4-producing CD4^+^ T-cells. There has been no effect on worm load, but there was a considerable drop in tissue egg burden, with a 71.3% in tissue egg burden and a % drop in female adult worm fecundity. The SjGST DNA vaccine delivered *via* the nanoparticle gene delivery system had the same anti-fecundity effect on female adult schistosomes as the conventional subunit vaccine with adjuvant, indicating that this DNA vaccine formulation is a promising candidate for anti-pathology and transmission-blocking applications	Mbanefo et al. (2015)
2	Alginate coated chitosan NPs	*S. mansoni*	Mice	Mice immunized with CpG-associated nanoparticles exhibited important modulation of the granuloma reaction. Orally NPs immunized mice from all the groups provided a substantial degree of defense against the risk of infection with *S. mansoni* worms, This suggests that chitosan plays a key role in generating a protective immune response. Mice vaccinated with SmRho plus antigen-based nanoparticles linked to CpG reduced granuloma by 38% and gave 48% protection against *S. mansoni* infection	Oliveira et al. (2012b)
3	SWAP-MSN	*S. mansoni*	Mice	During the 120-day experiment, mice were given only SWAP (as a negative control) showed no increase in IgG1 levels, indicating that they were only maintaining homeostatic levels. Mice given SWAP-loaded MSNs or SWAP combined with aluminum salt, on the other hand, had a significant increase in serum IgG1 levels, indicating that both formulations could stimulate the mice’s immune system against Schistosoma mansoni. It is worth noting that until 14 days after immunization, no significant differences in serum IgG1 levels were observed between the three groups. After 14 days, however, mice treated with SWAP-loaded MSNs had higher antigen levels than other groups (*p* > 0.5)	de Pádua Oliveira et al. (2016)
4	chitosan NPs loaded with plasmid DNA encoding Rho1-GTPase	*S. mansoni*	Mice	The result of this study shows chitosan NPs can significantly reduce liver pathology. Animals immunized with CH nanoparticles without DNA and challenged with cercariae had a 47% reduction in adult worm burden when compared to the saline control group	Oliveira et al. (2012a)
5	AuNHs-NH_2_-rSm29	*S. mansoni*	Mice	The AuNHs-NH2-rSm29-treated group had a higher level of protection (34%). In AuNRs-NH2-rSm29 immunization Th1 immunological response with increased IFN-ɤ production, mostly by CD4^+^ and CD8^+^ T cells, was found. *In vitro* study, these nanorods also activate dendritic cells, boosting MHCII and MHCI expression as well as IL-1 β n production in an NLRP3-, ASC-, and Caspase-1-dependent way	Assis et al. (2018)
6	PAMAM-Lys demdrimer	*S. japonicum*	Mice	PAMAM-Lys dendrimers are a new vaccine delivery vector that can boost DNA vaccine immunoreactivity and protect against *S. japonicum* infestation. Antibodies were significantly higher in PAMAM-Lys combined DNA vaccine-treated mice than in naked DNA vaccine-treated mice. The PAMAM-Lys vector induced an IgG2a-dominated antibody response as well as a significant increase in IL-2 and IFN-c production	Wang et al. (2014)

### Advantages and disadvantages of nanotechnology

A lot of research has been conducted on nanotechnology because nanoparticles with dimensions of 100 nm or fewer usually have a high specific zone, a high adsorption capacity, and a variety of particular physicochemical, optical, and electrical characteristics (Ghormade et al., 2011). Nano-loading of NPs in nano-drug delivery systems can alter the permeability, of the membrane and remove special big barriers, thereby endorsing straight drug diffusion and intracellular delivery (Sun et al., 2008; Kunzmann et al., 2011). A nano drug delivery system can therefore increase consumption levels and curative effects, and decrease drug side effects. Biosensors are an influential and creative analytical tool with admirable classifications, very sensitive, quick, simple, and low-cost. It can be used in the field of drug finding, diagnosis, biomedicine, food safety, and environmental monitoring (Ullah et al., 2020c). As a result, these devices have become extensively established and employed in a variety of biological disciplines, including cancer detection, diagnostic reagent creation, and gene therapy. The relatively high cost and adverse environmental impact of the most often utilized NPs, such as gold, iron oxide, or ZnO-like NPs, severely limit their agro-biotechnological uses (Sun et al., 2008; Tang et al., 2012). The biosensor is also not commercially available for the majority of infections; further research is needed to evolve the technology (Janissen et al., 2017; Ullah et al., 2020c).

## Future prospective

This review comprehensively documented the application of nanoparticles in schistosomiasis treatment, prevention, and diagnosis. Despite various treatments, option schistosomiasis is frequently seen in endemic as well as non-endemic areas. Further, the basic hurdle in schistosomiasis treatment is the low bioavailability of anti-schistosomal materials, the resistance of parasites due to misuse of drugs, and improper diagnosis. Researchers are working on the understanding treatment, diagnosis, prevention, and control of schistosomiasis. Unluckily there has not been any promising approach toward anti-schistosomal therapy. The involvement of scientists would confidently tackle the improvement of the treatment plans for this deliberating disease. Nanomedicine is an important application of nanotechnology for medical science for the last 20 years, which has grown as one of the most favorable techniques for the precision of conservative chemotherapies and diagnosis. The drug delivery system has the capability of administration of a low dose of drug and target-specific activity could be achieved by using nanotechnology. However, the fabrication and manipulation of nanomaterials in a repeatable and cost-effective way are still in their early stages. But it is expected that the application of NMs in schistosomiasis therapy will improve the current methods used in the detection, treatment, and control. The challenges involved in anti-schistosomal chemotherapy will be minimized by nanomedicine. The use of nanotechnology will increase bioavailability, decrease the quantity, toxicity, and side effects, and surge patient compliance. Hopefully, nanomedicine will deliver fairly improved and economical anti-schistosomal treatment practices than conventional chemotherapy, and probably will decrease the fiscal load of this tropical neglected disease. Therefore, nanotechnology approaches will encompass a wide range of solutions over conventional approaches in the upcoming days, for fast diagnosis, control, and prevention of this tropical neglected disease. It will be critical to discuss alternative ways to improve existing nanomedicine or nanotechnology approaches to improve schistosomiasis diagnosis and treatment.

## Conclusion

Schistosomiasis continues to increase globally in tropical regions. It upsets the world’s deprived countries where there is a lack of basic facilities like safe water, sanitation, and other hygiene environmental conditions. The increasing shortcomings in the use of PZQ and other commonly used diagnostic methods have paved toward the use of potential alternative drug therapies. In this analysis, we have focused on the current application of nanomaterials used in biomedicine. Because of their distinct properties, nanomaterials have attracted a great deal of attention, and they have been utilized in the improvement of diagnostic techniques, therapeutic targets, and schistosomiasis prevention and vaccine. NPs are novel drug delivery systems for PZQ, which is a powerful anti-schistosomal medication that may be employed against different stages of *Schistosoma*. Silver, gold, selenium, silica, liposome, etc. are considered potential nanomaterials used for the treatment, and as vaccine candidates against schistosomiasis. Nanoparticles are easy to develop, have low toxicity, improve drug bioavailability by solubility modification, and improve drug absorption across the biological barrier. Nanotechnology also improves the sensitivity and efficacy of diagnostic kits. Therefore, the combination of these nanomaterial products may change the existing situation of therapeutics, control, and medical diagnosis.

## Glossary

AChE: Acetylcholinesterase
AgNPs: Silver nanoparticles
AuNPs: Gold nanoparticles
BN: Boron nitride
BNNSs: Boron nitride nano spheres
Cur-GNPs: Curcumin loaded gold nanocapsules
DNA: Deoxyribonucleic acid
CTAB: Cetyltrimethylammonium bromide
DDS: Drug delivery system
ED95: Effective dose 95
ENMs: Engineered nanomaterial’s
GICA: Colloidal gold immunochromatography assay
Lip.PZQ: Liposomal-Praziquantel
LNCs: Lipid nanocapsules
LOXA: Liposomes entrapped oxamniquine
nAChR: Nicotinic type of acetylcholine receptor
NMs: Nanomaterials
NPs: Nanoparticles
M-PZQ: Market praziquantel
MFS-LNC: Miltefosine lipid nanocapusles
MSNs: Mesoporous silica nanoparticles
PZQ: Praziquantel
PZQ-Si: Praziquantel-mesoporous silica
PZQ-NLC2: Praziquantel-Nanostructured lipid carriers 2
PZQ-NLC4: Praziquantel- Nanostructured lipid carriers
ROS: Reactive oxygen species
S: Schistosoma
Se-NPs: Selenium-nanoparticles
SGTP1: Schistosome glucose transporter 1
SGTP4: Schistosome glucose transporter 4
SLNs: Solid nanoparticles
SLN-PZQ: Solid nanoparticles praziquantel
SWAP: Soluble worm antigenic preparation
VL: Visceral leishmaniasis
WHO: World health organization
ZnO-NPs: Zinc oxide nanoparticles
